# 421. Effect of SARs-Cov-2 mRNA Vaccination in Healthcare Workers with Household COVID Exposure

**DOI:** 10.1093/ofid/ofab466.621

**Published:** 2021-12-04

**Authors:** Laura Selby, Richard Starlin

**Affiliations:** University of Nebraska Medicine, Omaha, Nebraska

## Abstract

**Background:**

Healthcare workers have experienced a significant burden of COVID-19 disease. COVID mRNA vaccines have shown great efficacy in prevention of severe disease and hospitalization due to COVID infection, but limited data is available about acquisition of infection and asymptomatic viral shedding.

**Methods:**

Fully vaccinated healthcare workers at a tertiary-care academic medical center in Omaha Nebraska who reported a household exposure to COVID-19 infection are eligible for a screening program in which they are serially screened with PCR but allowed to work if negative on initial test and asymptomatic. Serial screening by NP swab was completed every 5-7 days, and workers became excluded from work if testing was positive or became symptomatic.

**Results:**

Of the 94 employees who were fully vaccinated at the time of the household exposure to COVID-19 infection, 78 completed serial testing and were negative. Sixteen were positive on initial or subsequent screening. Vaccine failure rate of 17.0% (16/94).

Healthcare workers exposed to household COVID positive contact

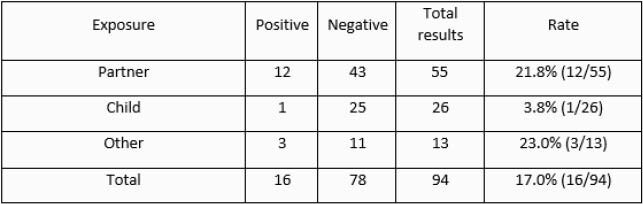

**Conclusion:**

High risk household exposures to COVID-19 infection remains a significant potential source of infections in healthcare workers even after workers are fully vaccinated with COVID mRNA vaccines especially those with contact to positive domestic partners.

**Disclosures:**

**All Authors**: No reported disclosures

